# Donor-Specific Transcriptomic Analysis of Alzheimer's Disease-Associated Hypometabolism Highlights a Unique Donor, Ribosomal Proteins and Microglia

**DOI:** 10.1523/ENEURO.0255-20.2020

**Published:** 2020-12-15

**Authors:** Sejal Patel, Derek Howard, Alana Man, Deborah Schwartz, Joelle Jee, Daniel Felsky, Zdenka Pausova, Tomas Paus, Leon French

**Affiliations:** 1 Krembil Centre for Neuroinformatics, Centre for Addiction and Mental Health, Toronto, Ontario M5T 1L8, Canada; 2 Victoria College, University of Toronto, Toronto, Ontario M5S 1K7, Canada; 3 Rotman Research Institute, Baycrest Centre for Geriatric Care, University of Toronto, Toronto, Ontario M6A 2E1, Canada; 4 Department of Psychology, University of Toronto, Toronto, Ontario M5S 3G3, Canada; 5 Faculty of Arts and Science, University of Toronto, Toronto, Ontario M5S 3G3, Canada; 6 The Hospital for Sick Children, University of Toronto, Toronto, Ontario M5G 1X8, Canada; 7 Department of Psychiatry, University of Toronto, Ontario M5S 3G3, Toronto; 8 Institute for Medical Science, University of Toronto, Toronto, Ontario M5S 1A8, Canada; 9 Campbell Family Mental Health Research Institute, Centre for Addiction and Mental Health, Toronto, Ontario M5T 1L8, Canada

**Keywords:** microglia, neurodegeneration, neuroinflammation, transcriptomics

## Abstract

Alzheimer’s disease (AD) starts decades before clinical symptoms appear. Low-glucose utilization in regions of the cerebral cortex marks early AD. To identify these regions, we conducted a voxel-wise meta-analysis of previous studies conducted with positron emission tomography that compared AD patients with healthy controls. The resulting map marks hypometabolism in the posterior cingulate, middle frontal, angular gyrus, and middle and inferior temporal regions. Using the Allen Human Brain Atlas, we identified genes that show spatial correlation across the cerebral cortex between their expression and this hypometabolism. Of the six brains in the Atlas, one demonstrated a strong spatial correlation between gene expression and hypometabolism. Previous neuropathological assessment of this brain from a 39-year-old male noted a neurofibrillary tangle in the entorhinal cortex. Using the transcriptomic data, we estimate lower proportions of neurons and more microglia in the hypometabolic regions when comparing this donor’s brain with the other five donors. Within this single brain, signal recognition particle (SRP)-dependent cotranslational protein targeting genes, which encode primarily cytosolic ribosome proteins, are highly expressed in the hypometabolic regions. Analyses of human and mouse data show that expression of these genes increases progressively across AD-associated states of microglial activation. In addition, genes involved in cell killing, chronic inflammation, ubiquitination, tRNA aminoacylation, and vacuole sorting are associated with the hypometabolism map. These genes suggest disruption of the protein life cycle and neuroimmune activation. Taken together, our molecular characterization reveals a link to AD-associated hypometabolism that may be relevant to preclinical stages of AD.

## Visual Abstract

**Figure:**
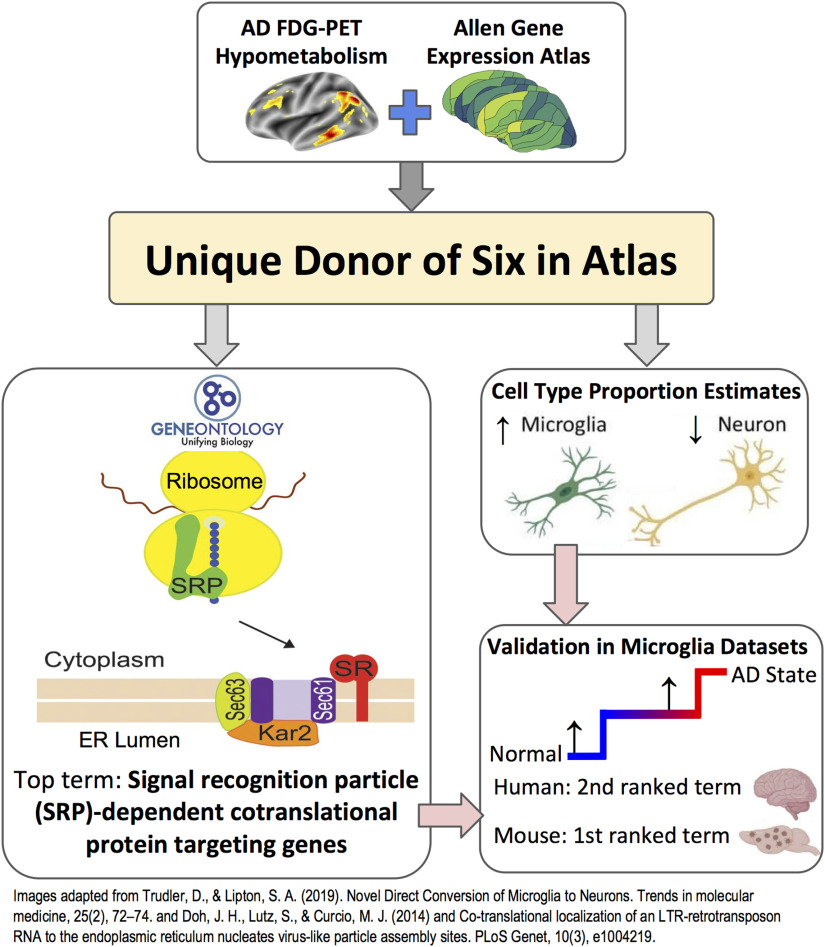

## Significance Statement

Fluorodeoxyglucose positron emission tomography (FDG-PET) is a frontline tool for the diagnosis of dementia. We sought to determine the molecular underpinnings of the metabolic signatures of Alzheimer’s disease (AD) revealed by FDG-PET. We found that of the six brains in the Allen Human Brain Atlas, a set of ribosomal proteins strongly aligned with the hypometabolism map in one of the six Atlased brains. While this brain was from a 39-year-old, it contained a neurofibrillary tangle in the entorhinal cortex. We observe changes in estimated neuron and microglia proportions that also suggest this individual had prodromal AD. In other studies, expression of the ribosomal genes increases across AD-associated microglial activation.

## Introduction

Alzheimer’s disease (AD), one of the most prevalent neurodegenerative diseases, is thought to affect ∼5% of those aged 60 years and above worldwide (Qiu et al., 2009). It is the most common form of dementia, which is clinically characterized by a severe decline in cognitive functioning and defined neuropathologically by the emergence and topographical progression of amyloid plaques, neurofibrillary tangles, and neuronal loss (Masters et al., 2015).

Currently, fluorodeoxyglucose positron emission tomography (FDG-PET) is a primary frontline tool for diagnosing dementia and its subtypes. FDG-PET uses a radioactive tracer, [18F] FDG-PET, to measure glucose metabolism within the brain (Friedland et al., 1983), with altered cerebral glucose metabolism indicating AD with high sensitivity and specificity (Mosconi et al., 2008). Importantly, hypometabolism patterns can be seen in at-risk individuals decades before the development of symptoms (Reiman et al., 2004; Mosconi et al., 2006; Langbaum et al., 2010; Landau et al., 2011; Chen et al., 2012). This timing supports the concept that AD exists on a spectrum or continuum of pathologies that includes stages of subtle cognitive decline, mild cognitive impairment, and dementia (Albert et al., 2011; McKhann et al., 2011). Despite the clear link between metabolic changes measured by FDG-PET and risk for AD, it remains unclear which etiopathological mechanisms are responsible for driving these changes.

Using the Allen Human Brain Atlas, we sought to characterize the pattern of regional hypometabolism found in patients with AD. By integrating this atlas with a meta-analytic map of FDG-PET differences, we identified genes with spatial expression patterns similar to that of the lower glucose metabolism in the AD brain. This transcriptomic approach was performed to identify consistent molecular markers of the FDG-PET pattern. To test the consistency of these markers, we performed the transcriptomic analysis within each of the six donors, which revealed a surprisingly strong association in a single donor. To better understand this signal, we examined cell-type proportion estimates. To validate this molecular and cell-type-specific marker of the FDG-PET pattern, we examined the relevant genes and cell-type in two datasets that profiled gene expression across AD-associated states.

## Materials and Methods

### Meta-analysis of Alzheimer’s FDG-PET studies

We performed a meta-analysis of FDG-PET studies that compared, at rest, Alzheimer’s patients with healthy controls. To compile a list of studies, a literature search was conducted on studies from January 1985 to January 2012. We used the following search query: [FDG-PET OR positron emission tomography OR fluorodeoxyglucose OR glucose metabolism] AND [dementia]. Studies were examined to fulfill the following criteria: (1) original research papers available in English (no case studies or reviews); (2) participants examined using [18F] FDG-PET at rest (no functional tasks); (3) AD patients compared with age-matched healthy controls; (4) clinical diagnosis of AD using NINCDS-ADRDA (McKhann et al., 1984) or DSM-IV (American Psychiatric Association, 1994) criteria; and (5) whole-brain analyses (no region-of-interest analyses) conducted in standardized stereotaxic space with available coordinates. Each article was read twice to determine whether the study met the inclusion criteria.

Coordinates of regional hypometabolism peaks from retained articles were used to create ALE maps using BrainMap’s GingerALE application (www.brainmap.org/ale; Eickhoff et al., 2009). This software assigns each voxel an activation likelihood estimate equal to the probability of at least one of the reported peaks of hypometabolism being located in that voxel (Turkeltaub et al., 2002). These voxelwise maps were clustered to find distinct anatomic clusters [min cluster extent = 500 mm^3^; false discovery rate (FDR) *q* = 0.05]. The identified clusters were then used to determine a threshold that marks which samples are inside regions of hypometabolism.

### Gene expression data

We used the Allen Human Brain Atlas to identify genes with spatial expression patterns similar to the FDG-PET hypometabolism map. This Atlas provides a comprehensive transcriptional landscape of the adult human brain (Hawrylycz et al., 2012). The Atlas was obtained from six individuals (five males, one female), with age ranging from 24 to 57 years. Custom 64K Agilent microarrays were used to assay genome-wide expression in 3702 spatially-resolved samples (232 named brain regions). We also used the RNA-sequencing datasets that were generated on the Illumina HiSeq2000 platform. These RNA-sequencing data were quantified with transcripts per million (TPM) and assayed a subset of anatomic structures from two of the six brains. The Allen Institute normalized the data and adjusted for array-specific biases, batch, and dissection method. Microarray probes were filtered using quality control data provided by Miller et al. (2014). After this filtering, 31,452 probes remained of the 58,692 on the microarray.

### Differential expression analyses

The Allen Human Brain Atlas gene expression data were first used at the sample and donor level to identify genes that are differentially expressed in the regions of hypometabolism identified by the ALE-based analysis. Expression values were mean-averaged for genes with multiple probes, resulting in 15,143 genes. This analysis was restricted to samples from the cerebral cortex, as marked by the Allen Human Brain Atlas annotations (allocortical regions, namely the hippocampal formation and piriform cortex, were excluded). For each donor and gene, expression values were compared between samples inside and outside of the hypometabolic regions using the Mann–Whitney *U* test. The Allen Institute provided MNI coordinates, which were used to map expression values into the voxel space of the meta-analysis. For analyses that spanned multiple donors, Fisher’s method was used to generate a single meta *p* value for each gene and direction (Fisher, 1925). We used the Benjamini–Hochberg FDR procedure for multiple test correction to adjust for the many tested genes (Benjamini and Hochberg, 1995).

### Gene Ontology (GO) enrichment analysis

The GO provides gene-level annotations that span specific cellular components, biological processes, and molecular functions (Ashburner et al., 2000). These annotations, defined by GO terms, were required to have annotations for 10–200 tested genes (6333 GO groups annotating 14,241 unique genes). To test for enrichment, we sorted the genes from the most overexpressed to underexpressed in regions of hypometabolism. Within this ranking, the area under the receiver operating characteristic curve (AUC) was used to test for GO terms that are enriched in either direction (overexpressed: AUC > 0.5, underexpressed: AUC < 0.5). The Mann–Whitney *U* test was used to determine statistical significance with FDR correction for the GO groups tested. We used GO annotations from the GO.db and org.Hs.eg.db packages in R, version 3.8.2, which were dated April 24, 2019 (Carlson, 2016a,b). We used the REVIGO tool to summarize many terms that were significant after correction (Supek et al., 2011). We used the default REVIGO parameters with uncorrected *p* values for the input GO groups and restricted this analysis to the biological process branch of the GO.

### Estimation of cell-type proportions

The Marker Gene Profile (MGP) tool was used to estimate cell-type proportions from the cerebral cortex expression profiles (Mancarci et al., 2017). This method uses the first principal component of the expression of cell-type-specific genes to estimate the relative abundance of a cell type. We used 21 top marker genes from a single cell study of the adult human temporal cortex (Darmanis et al., 2015; their Supplementary Table S3). This study used transcriptomic profiles to cluster cells into astrocyte, neuron, oligodendrocyte, oligodendrocyte precursor, microglia, and endothelial groups. These marker genes were used to calculate AUC values and estimate cell-type proportions with the MGP tool. Proportions were estimated separately for each donor across the same cortical samples used in the differential expression analysis.

### Single-cell RNA sequencing analysis of mouse microglia

Supplemental data from a single-cell RNA sequencing study of wild-type and AD transgenic mouse model (5XFAD) were used to examine gene expression in immune cell types (Keren-Shaul et al., 2017). Keren-Shaul and colleagues profiled trancriptomically 8016 immune cells from three wild-type and three 5XFAD mice and clustered these cells into 10 distinct subpopulations based on expression. Of these 10 clusters, three expressed microglia markers. Two of these microglia clusters contained cells primarily from 5XFAD and not wild-type mice and named them disease-associated microglia (DAM). For our analysis, we consider these clusters separately as different microglial states: normal, intermediate (Group II DAM), and AD associated (Group III DAM).

### Single-nucleus RNA sequencing analysis

Supplemental data from a single-nucleus RNA sequencing study of the human prefrontal cortex were used to examine differential expression across AD states in microglia. Specifically, for each gene, we extracted adjusted *p* values (IndModel.adj.pvals), mean expression, and fold changes (IndModel.FC) from Mathys et al. (2019; their Supplement Table 2). After quality control, Mathys et al. (2019) clustered the transcriptomes of 70,634 nuclei from 48 individuals into eight broad cell-type clusters. For this work, we focused on data from the 1920 microglia nuclei. The 48 participants in this study were classified into no (24), early (15), and late (9) AD pathology. To test for enrichment of our genes of interest, we sorted the genes from the most overexpressed to underexpressed for the differential expression results for no versus early pathology and early versus late pathology analyses. Within this ranking, the AUC was used to test for significantly enriched genes in either direction. We also used the mean expression to determine which genes increase in expression across the three pathology groups. For a given set of genes, the hypergeometric test was used to determine whether a greater number of genes increase across pathology than expected by chance.

### Code accessibility

Scripts for reproducing the analyses are publicly available online at https://github.com/leonfrench/AD-Allen-FDG and https://figshare.com/articles/dataset/Donor_specific_transcriptomic_analysis_of_Alzheimer_s_disease_associated_hypometabolism_highlights_a_unique_donor_microglia_and_ribosomal_prot eins/12233552 and as Extended Data 1.

10.1523/ENEURO.0255-20.2020.ed1 Extended Data 1 Zip file containing Rand Python source code to reproduce the analyses. Download Extended Data 1, ZIP file.

## Results

### Meta-analysis of FDG-PET studies of AD

**Figure 1.**
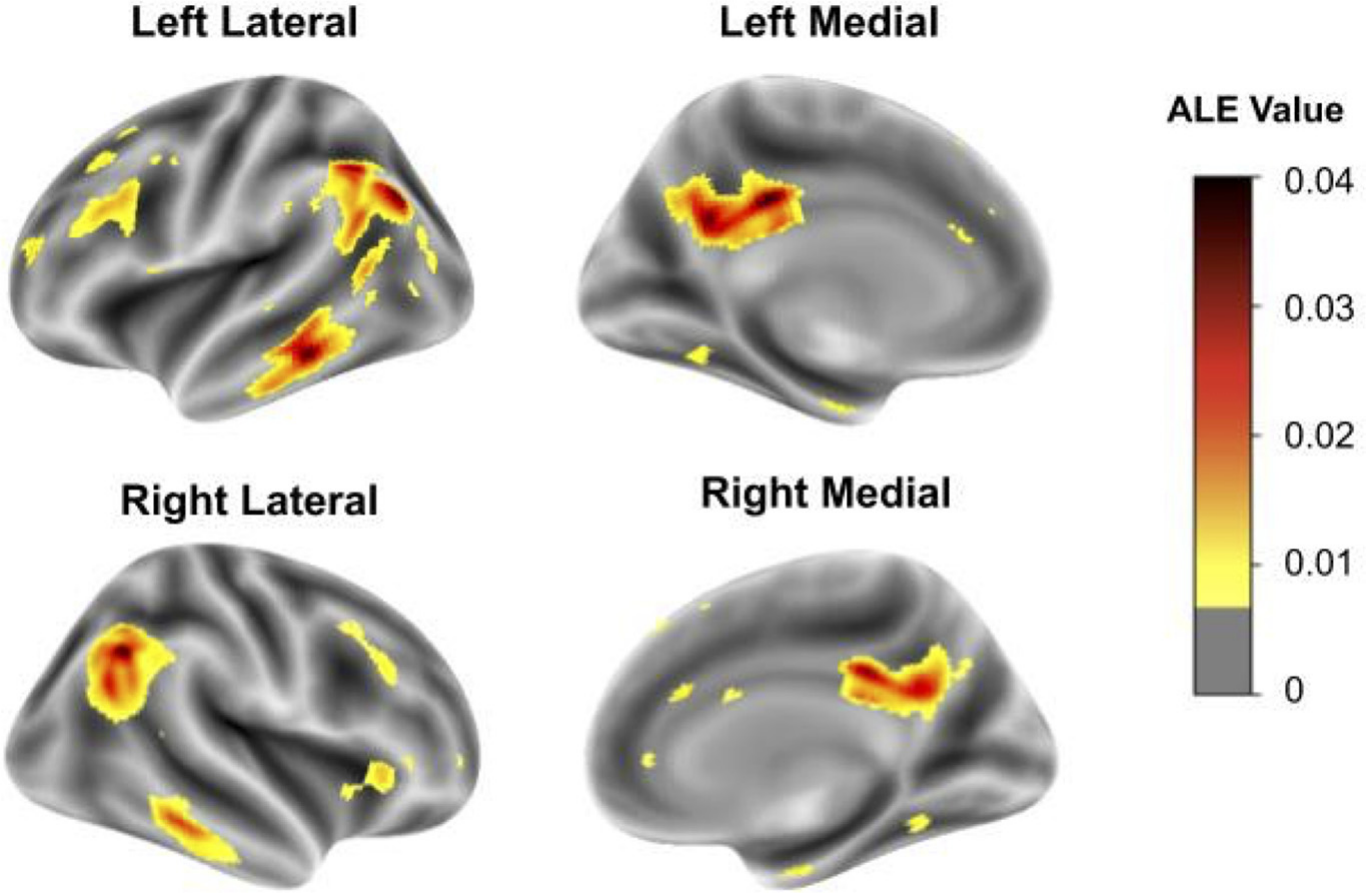
Cortical surface views of the ALE meta-analysis results. Regions where hypometabolism was not detected are transparent (ALE value of 0.006 or less). Lower glucose utilization (AD vs controls) ranges from low (yellow) to high (black).

Our literature search for FDG-PET studies identified 3229 titles. Screening of the abstracts yielded 230 relevant studies. Upon review of the full articles, 29 relevant studies remained. When two studies used the same patient population, one of the overlapping studies was excluded, resulting in a total of 27 studies yielding 33 independent samples with a total of 915 Alzheimer’s patients and 715 healthy controls (details in Extended Data Fig. 1-1). Activation likelihood estimation (ALE) meta-analysis of these studies identified the following cortical regions as showing (consistently) lower glucose metabolism in patients versus controls: posterior cingulate gyrus, middle frontal region, angular gyrus, and middle and inferior temporal regions. A cluster analysis revealed 23 clusters (min cluster extent = 500 mm^3^; FDR *q* = 0.05). A voxel-wise threshold of 0.006 was set to mirror this clustering map (Fig. 1) and was used to determine whether a given voxel was inside an AD-associated region of hypometabolism in subsequent transcriptomic analyses.

10.1523/ENEURO.0255-20.2020.f1-1 Figure 1-1 Details of the FDG-PET studies used in the meta-analysis. Download Figure 1-1, XLSX file.

### Many genes are differentially expressed in cortical regions with AD-associated hypometabolism

To identify molecular signatures underlying AD hypometabolism, we next performed a transcriptome-wide analysis to test for genes that correlate with the FDG-PET derived map. Using all six brains included in the Allen Atlas, we first identified the genes that were differentially expressed in the FDG-PET-defined hypometabolic regions of the cerebral cortex (one female and five males, aged 24–57 years). The number of cerebral cortex samples profiled by the Allen Institute ranged from 182 to 481 per donor; 5.9–9.9% overlapped with the hypometabolic regions. Of the 15,143 genes tested, 99 were significantly expressed at higher levels, and 51 at lower levels in these hypometabolic regions, after correction, across all donors. Substantial variability across the six brains in the Allen Human Brain Atlas has been previously noted both genome-wide and in the context of AD (French and Paus, 2015; Hawrylycz et al., 2015; Grothe et al., 2018; Ritchie et al., 2018). Given this variability, we then tested each brain separately. Strikingly, one brain drove the majority of the above atlas-wide signal for spatial expression overlap with the FDG-PET-derived map. In this brain (10 021/H0351.2002), 647 genes were differentially expressed, with 74% being expressed at lower levels in the hypometabolic regions. In the remaining five donor brains, differentially expressed genes were only found in the oldest donor (donor 12 876/H0351.1009, 57-year-old male). Taken together, our analysis of brain 10 021/H0351.2002 marks it as an outlier with hundreds of genes that align spatially with the patterns of lower glucose metabolism observed in patients with AD (vs controls).

### Brain-specific analyses point to a unique donor

We examined the demographic information and metadata of this donor to help understand the above observation. Brain 10 021/H0351.2002 was from a 39-year-old male African American individual. The postmortem interval was 10 h, the lowest of the six donors. In agreement, RNA integrity values (RINs) for this brain are higher than the other donors for all four regions assayed for RIN (frontal pole: 7.5, occipital pole: 7.1, cerebellum: 8.6, and brainstem: 7.3). As documented by the Allen Institute, this donor, like the others, had no known history of neuropsychiatric or neurologic conditions. The presence of a broad range of drugs was tested for in postmortem blood by the Allen Institute. In donor 10 021/H0351.2002, atropine, caffeine, lidocaine, and monoethylglycinexylidide were detected at levels usually not toxicologically significant. We note that monoethylglycinexylidide is a metabolite of lidocaine, an anesthetic and antiarrhythmic agent. Among the six donors, only 10 021/H0351.2002 tested positive for lidocaine and monoethylglycinexylidide. The included brains were also classified as “normal” by a radiologist or pathologist. While considered neurotypical, it was noted that 10 021/H0351.2002 contained a single neurofibrillary tangle in the entorhinal cortex. Neurofibrillary tangles in the hippocampus and entorhinal cortex are considered early events in AD progression (Guillozet et al., 2003). Neurofibrillary tangles were not found in the other five brains (three of which are older than this donor). The presence of a neurofibrillary tangle is a unique feature of this individual. The postmortem interval and RIN values suggest that tissue quality is not driving the Alzheimer’s-associated molecular patterns observed.

### ER translocation genes are enriched for overexpression in areas of Alzheimer’s-associated hypometabolism

**Figure 2.**
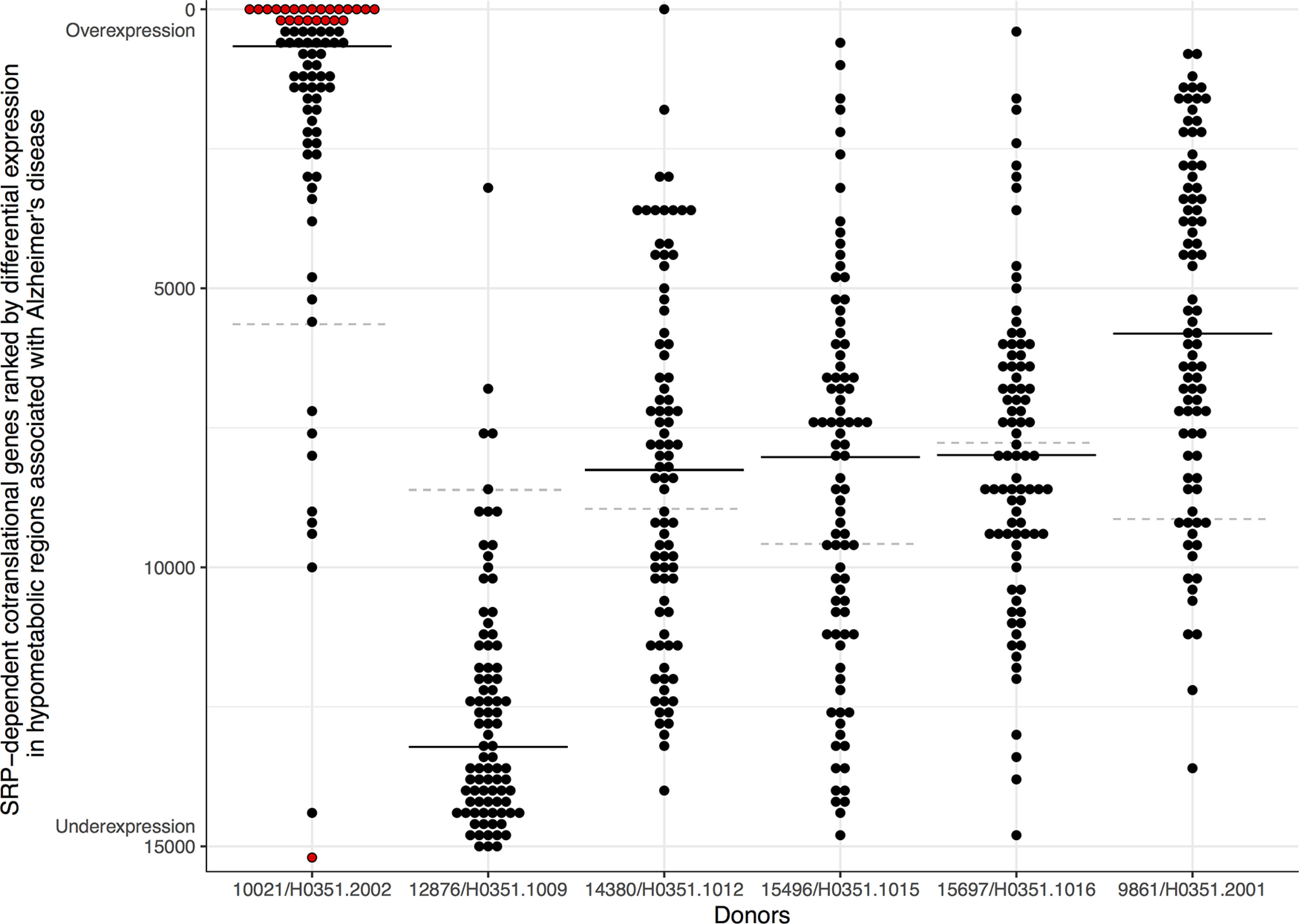
SRP-dependent cotranslational genes ranked based on differential expression in hypometabolic regions associated with AD. Genes are marked with dots, with the *y*-axis representing the genome-wide differential expression rank and ranges from overexpression (top) to underexpression (bottom). The black line marks the median expression rank of the SRP-dependent cotranslational genes. The dashed gray line marks the gene with the most stable expression between inside and outside of each donor’s hypometabolic regions. Red highlights genes that pass correction for multiple testing.

To distil the molecular results, we performed GO enrichment analysis on the transcriptome-wide results from donor brain 10 021/H0351.2002. In total, 215 GO groups were significantly enriched. Table 1 shows the top 10 GO terms enriched for genes upregulated in hypometabolic regions and Extended Data Table 1-1 contains complete enrichment results for all donors separately. Because of the high degree of overlap in gene membership among our top GO terms, we used REVIGO tool to summarize them (Supek et al., 2011). This tool removes redundant GO terms based on semantic similarity, providing a dispensability metric. Of the 98 biological process terms enriched for overexpression, three were assigned the lowest possible dispensability score of zero: SRP-dependent cotranslational protein targeting to membrane (GO:0006614, 87 genes, AUC = 0.874, *p*_FDR_ < 10^−28^), chronic inflammatory response (GO:0002544, 15 genes, AUC = 0.78, *p*_FDR_ < 0.05), and cell killing (GO:0001906, 94 genes, AUC = 0.60, *p*_FDR_ < 0.05). The strongest signal is from genes involved in SRP-dependent cotranslational protein targeting to membrane (Fig. 2). This process targets protein translocation to the endoplasmic reticulum via the signal-recognition particle (SRP). These genes are primarily components of the cytosolic ribosome and henceforth referred to as “ER translocation” genes. Six of these genes are found within the top 20 genes with higher expression in hypometabolic regions (*RPL34*, *RPL32*, *RPS27*, *RPS27A*, *RPL37A*, and *RPS15A*). In contrast, genes that are underexpressed in regions of hypometabolism are less significantly enriched for specific GO terms (lowest *p*_FDR_ = 7.3 × 10^−8^). However, these top terms contain more diverse themes (Table 1, bottom half), some of which have been previously implicated in AD. The most significant GO terms representing these themes are: “ubiquitin ligase complex”, “tRNA aminoacylation,” “ATPase activity, coupled,” “HOPS complex” (involved in endosomal vesicle tethering), and “microtubule organizing center part.” The ubiquitin-proteasome system has been linked to AD (Oddo, 2008). Of the four genes that encode ubiquitin, three with available data are strongly overexpressed in regions of hypometabolism in this brain. In summary, this enrichment analysis points to spatial differences in vesicle fusion, protein translation, targeting, and degradation.

**Table 1:** Top GO groups enriched for differential expression in areas of AD-associated hypometabolism in brain 10 021/H0351.2002

Name	Genes	ID	AUC	*p* value_FDR_
SRP-dependent cotranslational protein targeting to membrane	87	GO:0006614	0.874	1.35E-29
Cotranslational protein targeting to membrane	90	GO:0006613	0.865	2.07E-29
Protein targeting to ER	92	GO:0045047	0.847	2.86E-27
Cytosolic ribosome	87	GO:0022626	0.856	3.45E-27
Establishment of protein localization to endoplasmic reticulum	96	GO:0072599	0.828	1.66E-25
Structural constituent of ribosome	107	GO:0003735	0.794	1.05E-22
Ribosomal subunit	158	GO:0044391	0.737	1.01E-21
Nuclear-transcribed mRNA catabolic process, nonsense-mediated decay	104	GO:0000184	0.783	2.07E-20
Protein localization to endoplasmic reticulum	109	GO:0070972	0.765	9.44E-19
Cytosolic large ribosomal subunit	47	GO:0022625	0.894	6.73E-18

microtubule organizing center part	145	GO:0044450	0.395	0.00244
DNA-dependent ATPase activity	59	GO:0008094	0.33	0.00145
HOPS complex	13	GO:0030897	0.137	0.00135
ATPase activity, coupled	186	GO:0042623	0.396	0.00026
tRNA aminoacylation for protein translation	40	GO:0006418	0.268	9.84E-05
Amino acid activation	43	GO:0043038	0.275	8.24E-05
Aminoacyl-tRNA ligase activity	33	GO:0004812	0.243	8.24E-05
Cullin-RING ubiquitin ligase complex	111	GO:0031461	0.355	3.84E-05
tRNA aminoacylation	42	GO:0043039	0.259	1.91E-05
Ubiquitin ligase complex	195	GO:0000151	0.368	7.35E-08

10.1523/ENEURO.0255-20.2020.t1-1 Table 1-1 Complete GO enrichment results for all donors separately. Download Table 1-1, XLS file.

### Validation of ER translocation gene enrichment with RNA sequencing data

Focusing on donor 10 021/H0351.2002, the top-ranked GO group, “SRP-dependent cotranslational protein targeting to membrane”/“ER translocation,” contains genes that are involved in the targeting of proteins to the endoplasmic reticulum. Given the high and ubiquitous expression of ribosomal protein genes, the ER translocation signal may be because of ceiling effects induced by the dynamic range of microarray gene expression profiling. We tested for the association using RNA sequencing data to address this concern, which has a broader dynamic range. We again observe that the ER translocation genes are enriched (100 cerebral cortex samples, AUC = 0.733, *p*_FDR_ < 10^−9^). While limited in sample coverage for donor 10 021/H0351.2002, the RNA sequencing data validates the finding of differential expression of ER translocation genes.

### Estimates of cell-type proportions are disrupted in hypometabolic regions in brain 10 021/H0351.2002

To test whether regional transcriptomic differences might be because of cell-type proportions, we performed enrichment analyses of cell-type-specific marker genes based on the differential expression results. In the five brains, microglia marker genes were expressed at low levels in the hypometabolic regions (underexpressed; AUC = 0.1, *p*_FDR_ < 10^–8^) while astrocyte and neuron markers were expressed at high levels (overexpressed; AUC > 0.66, *p*_FDR_ < 0.05). In contrast, brain 10 021/H0351.2002 showed an opposite pattern of enrichment. Using the MGP (Mancarci et al., 2017) tool, which uses a more complex parametric method, we also observe an interaction between hypometabolic regions and brain 10 021/H0351.2002, whereby estimates of microglia proportions are higher inside hypometabolic regions in brain 10 021/H0351.2002 (five genes, *t* = 2.1, *p* = 0.033) and estimated proportions of neurons are lower (21 genes, *t* = −4.0, *p* < 0.0001).

### ER translocation gene expression is high in AD-associated microglia (DAM)

**Figure 3.**
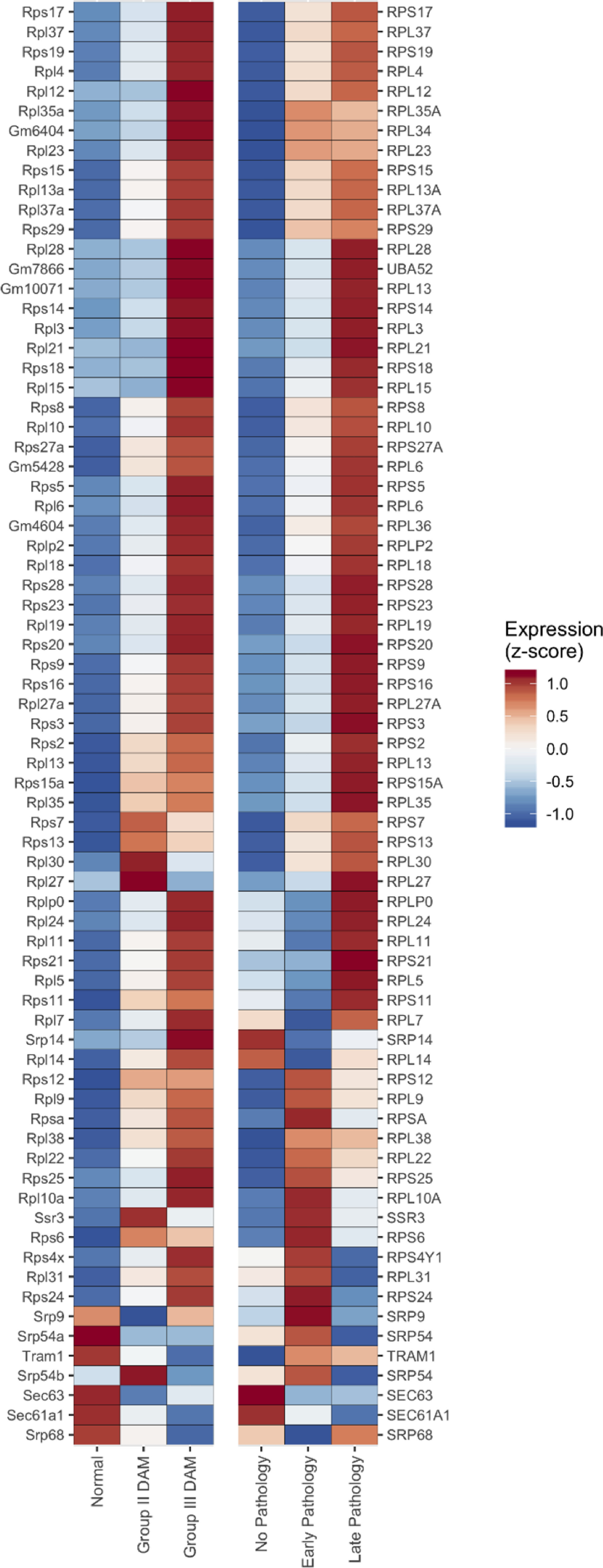
Heatmap of the ER translocation gene expression across three microglia cell clusters from the AD mouse model (left half) and AD pathology subgroups (right half). Expression for each gene is *z*-scored with high expression in red and low in blue. Genes are ordered based on hierarchical clustering using complete linkage (genes with similar expression across the mouse and human data are clustered together). Three human genes are duplicated because they have two homologous mouse genes (*RPL6*, *RPL13*, and *SRP54*). Human genes without homologous mouse genes are not displayed. Complete results spanning all GO groups are available as Extended Data Figure 3-1 (AD mouse model) and Extended Data Figure 3-2 (AD pathology subgroups). Data from other cell types in the AD pathology subgroups for the ER translocation genes are available in Extended Data Figure 3-3.

Based on the differential expression of microglia markers in donor 10 021/H0351.2002, we examined the ER translocation genes in microglia from an AD mouse model (Keren-Shaul et al., 2017). We tested whether the ER translocation genes increase in a stepwise pattern across the normal, intermediate, and full DAM clusters. For the 12,712 genes with data available, 6.5% monotonically increase in expression across these cell-type clusters that represent distinct states of AD-associated microglial activation. Of the 80 mouse homologs of the ER translocation genes, 75% increase in a stepwise fashion (Fig. 3, hypergeometric *p* < 10^–52^). Compared with all GO groups, this is the most significant enrichment (Extended Data Fig. 3-1). In this single-cell dataset, ER translocation genes are expressed in AD-associated microglia in a progressive pattern that suggests these genes increase with AD-associated microglial activation.

10.1523/ENEURO.0255-20.2020.f3-1 Figure 3-1 Complete GO enrichment results for step-wise increases across DAM clusters. Download Figure 3-1, CSV file.

### Expression of ER translocation genes is correlated with AD pathology

We next examined cell-type-specific transcriptomic data from postmortem human brain samples to reconnect the molecular markers with AD pathology. Specifically, using data from a single-nucleus study of the human prefrontal cortex, we next tested whether the ER translocation genes are differentially expressed across stages of AD pathology (Mathys et al., 2019). Guided by our findings in mice, we restricted our analyses to microglia. When comparing expression between no-pathology and early-pathology subgroups, we find that the ER translocation genes are enriched for higher expression in microglia from the early pathology individuals (79 genes, AUC = 0.716, *p *<* *10^–10^). For the comparison between early-pathology and late-pathology subgroups, the ER translocation genes are also enriched for higher expression in the late-pathology microglia (77 genes, AUC = 0.627, *p *<* *0.0005). Beyond these pairwise tests, we counted how many genes increase with disease progression. Broadly, for the 7319 genes with data available, the average microglial expression of 17.9% progressively increases across the pathologic groups. For the ER translocation genes, this proportion triples to 55.8% (Fig. 3, 43 of 77 genes progressively increase, hypergeometric *p* < 10^–13^). Compared with all GO groups, this is the second most significant group with the mostly overlapping set of cytosolic ribosome genes ranked first (Extended Data Fig. 3-2). While this relationship is strongest in microglia, astrocytes, oligodendrocytes, and their progenitor cells also have progressive increases of the ER translocation genes (proportion increased >36%, all *p* < 0.0002; Extended Data Fig. 3-3). In contrast, neither inhibitory nor excitatory neurons had progressively increased ER translocation gene expression across the pathologic groups. In this single-nucleus dataset, microglial expression of the ER translocation genes is correlated with AD progression.

10.1523/ENEURO.0255-20.2020.f3-2 Figure 3-2 Complete GO enrichment results for step-wise increases across the human AD pathology subgroups. Download Figure 3-2, CSV file.

10.1523/ENEURO.0255-20.2020.f3-3 Figure 3-3 ER translocation gene step-wise results for other cell-types across the human AD pathology subgroups. Download Figure 3-3, CSV file.

## Discussion

In this study, we projected the cerebral cortex’s transcriptome onto the spatial pattern of glucose hypometabolism found in AD cases. Our goal was to identify the molecular and cellular markers of this map. Of the six normal brains tested, only one demonstrated a robust spatial association between gene expression and the hypometabolism pattern. In support of this association, prior neuropathological examination of this individual found a neurofibrillary tangle. It is plausible that brain atlases seeking to assay the normal brain may contain samples from donors in the hypothetical stage of preclinical AD (Sperling et al., 2014). Our findings suggest that donor 10 021/H0351.2002 may have been on this path.

ER translocation genes, which encode proteins of the cytosolic ribosome and target protein translation to the endoplasmic reticulum, best align with the hypometabolic pattern in brain 10 021/H0351.2002. Using the transcriptomic data for this individual, we estimate a lower proportion of neurons and more microglia in hypometabolic regions. Beyond this single brain, we validate the associations between ER translocation genes and AD in microglia. Specifically, these genes have a staged expression pattern that increases across cellular and pathologic AD-associated states in human and mouse microglia. Together, these results that connect neuroimaging markers of AD with single-cell signals of neuroinflammation identify ER translocation machinery as an early dysregulated process in AD.

It is striking that the ER translocation GO group was the most significantly enriched set in our analysis of the 10 021/H0351.2002 donor brain and AD-associated microglia. It is known that cytosolic ribosome genes are strongly co-expressed (Lee et al., 2004). While we did not perform co-expression analysis, a change across this gene set will be easily detected with a pathway or GO analyses because of their high co-expression. This coherence is partly why it ranks above all other gene sets tested. Nonetheless, we note that a *RPL34* is a top-ranked gene, providing a strong signal at the level of single genes. To gauge the chance of this GO group being top-ranked in multiple studies, we checked whether the group is multifunctional or contains commonly differentially expressed genes. We found that this group ranked average in terms of multifunctional genes, relative to other groups (ranked 6848th of 11,404 GO groups; Gillis and Pavlidis, 2011). This group was also not top-ranked in any of the 635 studies systematically examined in a broad study of differential gene expression predictability (Crow et al., 2019). More directly, the ER translocation genes are stable, with a below-average prior probability of differential expression (ER translocation genes median = 0.246, remaining genes = 0.562, Mann–Whitney *U* test *p* < 10^–9^). Therefore, while ER translocation genes are strongly co-expressed, the prior likelihood of the ER translocation genes being differentially expressed is low.

The ribosome and protein synthesis have been previously associated with mild cognitive impairment and AD (Sajdel-Sulkowska and Marotta, 1984; Langstrom et al., 1989; Ding et al., 2005; Hernández-Ortega et al., 2016). Pathologic tau has also been shown to determine translational selectivity and co-localize with ribosomes (Meier et al., 2016; Koren et al., 2019). Beyond the ER translocation genes, we note other GO groups with functional relevance. For example, “chronic inflammatory response” and “cell killing” genes were enriched for overexpression in the hypometabolic regions in brain 10 021/H0351.2002. In the other direction, the genes in the homotypic fusion and protein sorting (HOPS) complex are underexpressed in hypometabolic regions in brain 10 021/H0351.2002. This complex contains vacuole sorting genes and regulates autophagosome-lysosome fusion (Balderhaar and Ungermann, 2013). The top two most underexpressed gene sets in the hypometabolic regions are “ubiquitin ligase complex” and “tRNA aminoacylation.” While ubiquitin ligase complex genes are underexpressed, genes encoding ubiquitin are overexpressed in the hypometabolic regions in brain 10 021/H0351.2002. In summary, analysis of this single brain identifies genes that function in the protein life cycle and neuroinflammation, which are known to be disrupted in AD (Heneka et al., 2015; Gadhave et al., 2016; Cheng et al., 2018).

Intriguingly, other studies have associated the ER translocation genes with neurodegeneration. In a recent postmortem study of two cohorts, the ER translocation genes were strongly downregulated in brain samples from Parkinson’s disease cases when compared with controls (Nido et al., 2020). While this contrasts our findings of upregulation, in the context of AD, two recent studies have also highlighted the ER translocation genes. First, an analysis of the Alzheimer’s brain transcriptome found that these genes are upregulated in Caribbean-Hispanic AD cases but not non-Hispanic whites (Felsky et al., 2020). The authors of this study speculate that the SRP-dependent protein targeting genes relate the gingipain hypothesis of AD causation that implicates *Porphyromonas gingivalis* (Dominy et al., 2019). A second study supports this connection by showing that the ER translocation genes are upregulated in cortical samples with detected *P. gingivalis* sequences and are enriched for the arginine and lysine residues that the gingipain proteases cleave at (Patel et al., 2020). By performing neuroanatomical analyses, this study also discovered that the ER translocation genes are highly expressed in hypothalamus, cholinergic neurons, and the basal forebrain. This spatial signature may explain early cholinergic degeneration and sleep disruptions in AD. Together, our findings and these studies that implicate the same genes promote ER translocation as an underlying disease mechanism that connects the cholinergic and gingipain hypotheses of AD causation.

In conclusion, the hypometabolism pattern that marks AD was correlated with the expression of genes encoding ribosomal ER translocation proteins. This association was observed in the brain of a 39-year-old that contained a neurofibrillary tangle in the entorhinal cortex. In this brain, the estimated proportion of microglia was higher in the hypometabolic regions. We speculate that this individual may have been in the hypothesized preclinical stage of AD that may last decades (Sperling et al., 2011). In AD-associated microglia obtained from the cortex of 48 individuals with a broad range of AD pathology, we extend these findings at the cellular level to show expression of the ER translocation genes progressively increases with AD pathology. This is most pronounced in microglia from individuals with early pathology. Our transcriptomic analysis of AD-associated hypometabolism warrants further study of ribosomes, the protein life cycle, and neuroimmune activation in models of early AD.

## Synthesis

Reviewing Editor: Francisca Bronfman, Universidad Andrés Bello Decisions are customarily a result of the Reviewing Editor and the peer reviewers coming together and discussing their recommendations until a consensus is reached. When revisions are invited, a fact-based synthesis statement explaining their decision and outlining what is needed to prepare a revision will be listed below. The following reviewer(s) agreed to reveal their identity: Paola Garcia-delaTorre.

Publish as it is.

## Author Response

The authors perform a systematic meta-analysis of FDG-PET in Alzheimer’s

disease studies to identify shared hypometabolism areas among AD

patients. Comparison with the donor brains in the Allen brain atlas

revealed one outlier donor that matched the hypometabolism pattern

yielding transcriptomic data relevant to early AD markers. Follow-up

analyses using publicly available datasets of single-cell RNA sequencing

in a mouse AD model and human AD studies support the role of microglia

dysfunction in early AD and suggest possible roles in ER translocation

for translation of membrane proteins.

General comments and concerns;

The main strength of the paper is that the follow-up analyses supported

the identification of ER translocation GO terms in the initial donor.

However, with the reliance of those initial findings on a donor where AD cannot be confirmed, it is necessary to make sure other possibilities are accounted for. A comparison with different cell types in the AD

datasets as well as comparisons with other neurodegenerative diseases, such as Parkinson’s disease, should be included.

To support further our initial findings, we have included an analysis of other brain cell

types. This is provided as Extended Data Figure 3-3 and is described in the last

paragraph of the Results section.

To determine more broadly if the ER translocation genes are relevant, we reviewed the

literature to identify studies highlighting these genes. This revealed interesting

connections in the context of Alzheimer’s and Parkinson’s disease. We now review and

compare our findings with these results in the last Discussion paragraph.

In the same line, the GO enrichment analysis on the transcriptome-wide

results was done in only one donor brain 10021/H0351.2002 ("To distill

the molecular results we performed GO enrichment analysis on the

transcriptome-wide results from donor brain 10021/H0351.2002.").

However, in Figure 2 appears that the authors had done the analysis for

all donors but discussed the results only for this particular one.

This is correct. We completed the analysis for all donors initially but only highlighted

brain 10021/H0351.2002. This is because this brain had the highest number of

differentially expressed genes. To improve clarity and transparency, we now include all

the - per brain - GO results as Extended Data Table 1-1 for interested readers.

However, donor 12876/H0351.1009 seems to have a different overall

expression than the remaining four donors, but no explanation is given

on why this is not important or relevant. Only by reviewing the title of

the article, it seems logic on how the paper became a report on only

donor 10021/H0351.2002 which appears by reading the results was randomly

found when using the Allen Atlas for the genetic analysis.

Donor 12876/H0351.1009 seems to have a different overall expression pattern only

when viewed in the light of the FDG-PET map. To our knowledge, none of the many

previous analyses of the Allen Human Brain Atlas has singled out this donor. In this

respect, it could be considered a random or chance discovery as most analyses that

use this Atlas do not perform donor-specific tests. We also did not expect such large

variations across the donors. Only upon further investigation did we discover that

12876/H0351.1009 was unique through the lens of gene expression, as well as in other

aspects.

Beyond our title, we also note the Result heading "Brain specific analyses point to a

unique donor" that helps describe our findings. However, we agree that the narrative

could be improved for clarity. Therefore, we have added several passages throughout

the manuscript to better explain the flow of analyses and how they support our

conclusions.

We believe that the pattern of the ER translocation gene expression that aligns with the

hypometabolism map is important. Our speculative explanation is that this individual

may have been at a stage of preclinical AD. However, this is a cautious observation

based on very limited (clinical) information that is available for this individual. To avoid

over-interpretation of incomplete pathological data, we do not spend a significant

amount of time describing features of this donor’s brain in the manuscript.

Also, to clarify for the online readers of this response, our analysis was transcriptomic

and not genetic as we did not examine genetic variation. In addition, six brains are

profiled in the Atlas, leaving five remaining donors (not four).

Overall, although the study is interesting, it is confusing. In general,the manuscript would benefit from re-organization, so it does not appear

a mix of several individual reports. The link between studies

(voxel-wise meta-analysis, gene identification using the Allen Human

Brain Atlas, and the use of transcriptomic data) is never there. It

looks like a mixture of papers with different approaches and no common goal. The results are vaguely related, and the discussion does not help

in untangling the former.

We agree that our switch from a spatial marker to a cell-type and molecular marker may

confuse readers. To improve the flow of our study and reduce confusion we have added

a graphical abstract, moved a section, and added text throughout. We have also added

a paragraph to the Discussion section to connect our findings to other studies better.

We agree that the FDG-PET voxel-wise meta-analysis could stand alone in its own

paper but felt this is not of broad interest given that this pattern is well known and used

clinically.

Many groups have performed similar analyses that relate neuroimaging findings with the

Allen Human Brain Atlas (Grothe et al., for example). These investigations motivated

our analysis of the FDG-PET pattern. Most of these studies stop after this step, but our

findings in a single brain that contained a neurofibrillary tangle motivated follow-up in

the mouse microglia transcriptomic datasets. To prevent confusion, like our cell-type

specific results, we note that gene identification using the Allen Human Atlas used

transcriptomic data.

Specific comments or questions for authors,

1-Why was the DSM-IV not considered as an inclusion criterion for

clinical diagnosis for AD when the years of publication are in

accordance with DSM-III and IV?

We thank the reviewers for noticing this. Upon looking back, we discovered and

corrected this inadvertent typographical error. Specifically, our meta-analysis included

two publications that used DSM-IV and none that used DSM-III for clinical diagnosis.

2-Why January of 2012? It is not clear how or why you chose 1985 to 2012

as the time frame for the meta-analysis. Understandably, 1985 would

guarantee the use of FDG-PET for glucose metabolism, but why 2012?

The FDG-PET meta-analysis was performed earlier due to the timing of this research.

We considered updating this analysis, but new FDG-PET case-control Alzheimer’s

disease studies were limited. We suspect this is due to its transition into a clinical

diagnostic tool.

3-Is there a reason for using the Allen Human Brain Atlas and not the

Aging, dementia, and traumatic brain injury dataset?

The Aging, Dementia and Traumatic Brain Injury Study dataset is an impressive

resource that we plan to use for future analyses. In the context of this work, we note that

it profiles only four brain regions that are much coarser than the hundreds in the Allen

Human Brain Atlas.

Our findings of higher estimates of microglia proportion in brain 10021/H0351.2002 led

us to investigate disease-associated microglia (DAM). Upon finding expression

differences in microglia in this mouse model, we tested for expression changes in

human microglia. Because bulk tissue was profiled in the Aging, Dementia and

Traumatic Brain Injury Study, only 5-10% of the assayed cells would be microglia.

Although methods are available to estimate cell-type proportions, we decided to use the

single nucleus dataset to gauge more directly the expression of ER translocation genes

in microglia from Alzheimer’s disease cases. Nonetheless, we have performed some

preliminary analyses in this dataset, and are continuing to examine it in the context of

our results reported here.
